# NGF, EPO, and IGF-1 in the Male Reproductive System

**DOI:** 10.3390/jcm13102918

**Published:** 2024-05-15

**Authors:** Chryssa Metallinou, Chrysovalanto Staneloudi, Konstantinos Nikolettos, Byron Asimakopoulos

**Affiliations:** 1 Laboratory of Physiology, Faculty of Medicine, School of Health Sciences, Democritus University of Thrace, 69100 Alexandroupolis, Greece; cmetalli@mbg.duth.gr (C.M.); knikolet@med.duth.gr (K.N.); 2 Laboratory of Exercise Physiology and Biochemistry, Department of Physical Education and Sport Science at Serres, Aristotle University of Thessaloniki, 54124 Thessaloniki, Greece; cstanelo@phed-sr.auth.gr

**Keywords:** nerve growth factor (NGF), erythropoietin (EPO), insulin-like growth factor-I (IGF-1), sperm motility, sperm vitality, male infertility

## Abstract

Several studies have demonstrated interesting results considering the implication of three growth factors (GFs), namely nerve growth factor (NGF), erythropoietin (EPO), and the insulin-like growth factor-I (IGF-1) in the physiology of male reproductive functions. This review provides insights into the effects of NGF, EPO, and IGF-1 on the male reproductive system, emphasizing mainly their effects on sperm motility and vitality. In the male reproductive system, the expression pattern of the NGF system varies according to the species and testicular development, playing a crucial role in morphogenesis and spermatogenesis. In humans, it seems that NGF positively affects sperm motility parameters and NGF supplementation in cryopreservation media improves post-thaw sperm motility. In animals, EPO is found in various male reproductive tissues, and in humans, the protein is present in seminal plasma and testicular germ cells. EPO receptors have been discovered in the plasma membrane of human spermatozoa, suggesting potential roles in sperm motility and vitality. In humans, IGF-1 is expressed mainly in Sertoli cells and is present in seminal plasma, contributing to cell development and the maturation of spermatozoa. IGF-1 seems to modulate sperm motility, and treatment with IGF-1 has a positive effect on sperm motility and vitality. Furthermore, lower levels of NGF or IGF-1 in seminal plasma are associated with infertility. Understanding the mechanisms of actions of these GFs in the male reproductive system may improve the outcome of sperm processing techniques.

## 1. Introduction

Growth factors (GFs) are naturally occurring substances, usually signaling proteins, that stimulate a variety of cell functions such as cell growth, proliferation, differentiation, inflammation, and tissue repair. Growth factors bind to specific receptors on the surface of their target cells and typically influence the behavior of neighboring cells in a paracrine or autocrine manner. It was previously shown that GFs have receptors in the reproductive tissues [1] and are therefore involved in the reproductive processes. Moreover, the beneficial effects of adding GFs on sperm parameters have been reported [2].

Among the GFs, nerve growth factor (NGF), erythropoietin (EPO), and insulin-like growth factor-I (IGF-1) have taken significant attention as several studies have demonstrated interesting results considering their implication in the physiology of male reproductive functions. NGF and EPO have long been recognized for their diverse roles in neuronal development and hematopoiesis, respectively. However, their implications in male reproductive function have only recently become a focal point of research interest. In parallel, IGF-1 has garnered attention for its multifaceted actions in modulating growth hormone activity and cellular differentiation, including its influence on male reproductive physiology.

This review provides insights into the effects of NGF, EPO, and IGF-1 mainly on the male reproductive system of humans with references to other species. In particular, emphasis will be placed on understanding how these GFs impact sperm motility and vitality, as a major problem concerning male infertility and the outcome of assisted reproduction techniques (ART). Additionally, other key aspects of male reproductive health, such as spermatogenesis, sperm function, and overall reproductive physiology will be discussed.

Further, this comprehensive review aims to synthesize existing research findings to explore expression patterns of NGF, EPO, IGF-1, and their receptors in male reproductive tissues across various species, with a primary focus on humans, in order to elucidate their contributions to male reproductive physiology. Furthermore, the potential therapeutic implications of these GFs are assessed for diagnosing and treating male infertility. Moreover, an attempt is made to identify gaps in current knowledge and propose future research directions to enhance understanding of how these GFs influence male reproductive health.

To ensure comprehensive coverage of relevant literature, a search of electronic databases was conducted, including PubMed, Web of Science, Embase, Scopus, and Google Scholar. The search was limited to peer-reviewed articles published in English up to the present date. The search was conducted using combinations of keywords related to “NGF”, “EPO”, “IGF-1”, “male reproductive system”, “sperm, “sperm motility”, “sperm vitality”, “infertility”, and “ART”. Additionally, reference lists of relevant articles and reviews were manually searched to identify further studies. The references of included articles were also studied. The eligibility criteria for inclusion in this review were studies focusing on the effects of NGF, EPO, and IGF-1 on the male reproductive system, particularly sperm motility and vitality.

Through a comprehensive analysis of the literature, this review aims to enhance our understanding of the intricate roles of GFs in male reproductive physiology and provide insights into its clinical relevance in the diagnosis and management of male infertility.

## 2. Nerve Growth Factor (NGF)

### 2.1. Introduction

Neurotrophins (NTs) belong to a growth factor family that regulates the function, growth, differentiation, and survival of vertebrate’s peripheral and central nervous systems [3,4,5]. The family of neurotrophins includes nerve growth factor (NGF), brain-derived neurotrophic factor (BDNF), neurotrophin 3 (NT-3), and neurotrophins 4/5 (NT-4/5) [6]. They are collectively named NTs, as they originate from a common ancestral gene and have significant structural and sequence similarities [7,8]. In addition to the nervous system, NTs are expressed in non-neuronal tissues such as the cardiovascular, immune, endocrine, and reproductive systems where it seems that they regulate the development and functioning of the cells [9].

NGF, an insulin-like protein, is the first discovered and probably the most extensively studied member of the family [10]. It acts as the most important neurotrophin in the mammal’s central and peripheral nervous, immune, and endocrine systems [11,12,13,14]. NGF possesses a tertiary structure based on three cysteine disulfide clusters and two pairs of twisted, antiparallel β-strands [15]. Its biological effects are mediated by the specific high-affinity protein tyrosine kinase Trk receptor (TrkA) and the low-affinity p75 receptor [16,17,18,19,20].

The high-affinity receptor induces the activation of mitogenic p38-mitogen-activated protein kinase (MAPK) and phosphatidylinositol 3-kinase (PI3K) pathways, causing actions such as growth, maturation, differentiation, and escape of apoptosis [21]. The low-affinity receptor p75, which serves as a pan-neurotrophin receptor, plays an important role in regulating cell death and apoptosis and promoting cell survival [22,23]. P75 can activate, autonomously, the nuclear factor kappa B (NF-kappa B), Akt, and c-Jun N-terminal kinase (JNK) pathways [22] or, as it is suggested when p75 and TrkA receptors are co-expressed, p75 can increase NGF-mediated TrkA activation [24,25]. It seems that NGF binding to p75 is essential to support TrkA activation in response to low levels of NGF [26,27]. These interactions between the TrkA and p75 pathways appear to be important for the actions of NGF on various cells [22].

### 2.2. NGF in the Male Reproductive System of Animal Models

#### 2.2.1. Expression Patterns of NGF and Its Receptors across Species

The finding that NGF and its receptors are expressed in male reproductive tissues raised many questions about its potential actions; thus, several studies were conducted in a variety of animal models. Initially, the presence of NGF and TrkA had been established in the testis and epididymis of mouse and rat [28]. The aforementioned finding led to new studies that are summarized in Table 1.

In mouse testis, it was reported that possible sources of NGF production were the Leydig, the peritubular myoid, and the Sertoli cells, but not the germ cells. The receptor TrkA was found exclusively in nongerm cells whereas the p75 gene was temporarily expressed during the germ cell development, and its mRNA expression was induced in cultured Sertoli cells and peritubular myoid cells, by NGF [29].

ChunMei et al., 2005, discovered that NGF was expressed in the testis of adult male rats, in particular in Leydig cells and primary and pachytene spermatocytes [30]. TrkA immunoreacted only to elongate spermatids, and p75 showed positive immunostaining in Sertoli and Leydig cells, the pachytene spermatocytes, and elongate spermatids. In the same study, immunoreactions for the NGF system were detected in epithelial cells of efferent and deferent duct, in the epididymis, in columnar secretory epithelium lines of the seminal vesicles, in the prostate, and in the coagulating gland.

Furthermore, increased immunoreactivity of NGF and its receptor TrKA was observed by Artico et al., 2007, in the testes of sexually mature male rats, untreated or treated with lonidamine, an antispermatogenic drug [31]. Remarkably, p75 was moderately expressed in all germ cells of treated rats and weakly expressed in the seminiferous tubules of untreated animals. 

Perrard et al., 2007 investigated the effect of bNGF on spermatogenesis and the presence of the NGF system in the testis sections, in Sertoli cells, germ cell fractions of rats, as well as in germ cell and Sertoli cell co-cultures [32]. NGF was detected in germ cells ranging from pachytene spermatocytes to spermatids, while TrkA and p75 were found in both Sertoli cells and germ cells, suggesting the possibility of an auto/paracrine mechanism, where NGF regulates the second meiotic division of rat spermatocytes in vivo.

Another interesting finding, by Levanti et al., 2006, was that the distribution pattern and intensity of immunoreaction of TrkA and p75 were slightly different in adult and newborn rats [33]. In testicles of adults, TrkA was expressed in spermatozoa and spermatids while p75 was expressed in spermatogonia. In newborn rats, TrkA immunoreactivity was found in the Leydig cells, whereas p75 was observed in a cellular layer that surrounds the seminiferous tubules. NGF was identified in the Leydig cells and in germinal cells at all stages, from primary spermatocytes to mature spermatozoids. The authors hypothesize that an autocrine and/or paracrine loop occurs in the seminiferous tubules of rat testicle.

In the adult male Japanese monkey (*Macaca fuscata*), immunohistochemistry showed that NGF and its receptors are located in Leydig cells, Sertoli cells, and spermatogonia at various stages. Signals for these proteins were also recognized in epithelial cells and stromal tissues of the caudal epididymidis, in addition to the seminal vesicle [34].

In ejaculated bovine sperm, both NGF and its receptor TkKA were detected [35]. NGF immunoreactivity was localized at the sperm head and tail, whereas TrkA was identified in the acrosomal cap, nucleus, and tail regions, indicating the possible effect of NGF on the differentiation, maturation, and movement of the spermatozoa [35].

Jin et al., 2010, in experiments with spermatozoa of golden hamsters, confirm the presence of NGF in Leydig cells [36]. A positive signal for NGF was also recognized in pachytene and preleptotene spermatocytes and elongated spermatids. TrkA was localized only in Sertoli cells, and p75 showed ubiquitous distribution in testis.

In rabbit’s male sex organs, the NGF system was detected in the testis, prostate gland, and seminal vesicle, and the highest levels of NGF and TrKA transcripts were found in the prostate [37].

In Llamas (*Lama glama*), NGF immunolabeling was positive for the prostate, while TrKA was detected in epithelial and muscular cells of the testis, epididymis, bulbourethral glands, and prostate. In addition, TrKA was observed in epididymal sperm, and NGF was located together with its high-affinity receptor in the middle piece of ejaculated and acrosome-reacted (AR) sperms, demonstrating that NGF could originate from seminal plasma [38].

According to the above information, it seems that the localization of NGF and its receptors depends on specific phenotypes of animal species [39] and appears to change with testicular development [40,41].

#### 2.2.2. Role of NGF in the Male Reproductive System

Regarding the role of the NGF system in the male reproductive system, studies mainly in animal models showed that NGF promotes spermatogenesis, acts as a potential regulator of meiosis [32,42], and may improve the formation and maintenance of the testicular seminiferous epithelium [41]. Further, it may play a significant role in early testicular development and adult testes morphology [43].

The significant role that NGF plays in the male reproductive system results from its interaction with its receptors, TrKA and p75. In animal models, NGF-induced TrkA receptor stimulation triggers the activation of pathways such as the Ras/MAPK, PI3K/Akt-mTOR, and phospholipase C (PLC) pathways. This action promotes cell survival and modulates sperm functions like survival, cell proliferation, and the acrosome reaction [44,45,46]. Mice that are missing NGF [47] or TrkA genes [9,48] do not survive after their birth.

On the other hand, p75 acts as a cell death receptor, and its activation affects capacitation, sperm motility, and apoptosis [44,45]. The actions of NGF seem to depend on the balance between its two receptors and which receptor pathway is activated, p75 pro-apoptotic or TrkA pro-survival. The balance seems to be determined by the ratio of p75 to TrkA [44] and probably regulates the cell fate, meaning sperm survival or apoptosis. Physiological NGF levels likely bind primarily to TrkA, supporting sperm, while excessive NGF levels associated with pathology may recruit p75, adversely affecting sperm traits. In the absence of TrkA, p75 serves as a selector for optimal sperm quality, by inducing apoptosis. This apoptotic process plays a crucial role in both the programmed aging of ejaculated sperm and the elimination of defective sperm generated during spermatogenesis and differs from the apoptotic processes of somatic cells [44].

Another issue of research interest is the question of the effects of NGF on sperm function, mainly mobility and vitality, with the results giving notable findings.

In bovines, exogenous NGF increased sperm viability and apoptosis but did not affect sperm mitochondrial activity, intracellular calcium concentrations, or the acrosome reaction, signifying that the NGF system may play significant roles in the regulation of sperm physiology but does not directly affect the sperm capacitation [35].

In golden hamsters, NGF treatment increased sperm motility and the acrosome reaction in a time- and dose-dependent manner [36]. The authors supported the notion that NGF plays an integral role in sperm motility by activating the MARK signaling pathway.

Sari et al., 2021, observed that the addition of human NGF to cryopreserved llama sperm improves sperm quality by promoting motility and maintaining viability and mitochondrial activity [49].

**Table 1:** Expression of the NGF system in the male reproductive tract.

References	Expression of NGF/Receptors	Cell-Type/Ort	Species
[28]	NGF/TrkA	Testis and Epididymis	Mouse and Rat
[29]	NGF	Leydig and Sertoli cells and peritubular myoid cells, but not the germ cells	Mouse
	TrkA	Nongerm cells	
	p75	Sertoli cells, peritubular myoid cells/temporarily expressed during the germ cell development	
[30]	NGF	Leydig cells, primary spermatocytes, and pachytene spermatocytes	Adult rats
	TrkA	Elongate spermatids	
	p75	Sertoli cells, Leydig cells, pachytene spermatocytes, and elongate spermatids	
[31]	NGF, TrKA	Seminiferous tubules	Rat
	p75	Tubules and in all the germ cells	
[32]	NGF	Germ cells from pachytene spermatocytes and spermatids	Rat
	TrkA, p75	Sertoli cells and germ cells	
[33]	NGF	Leydig cells and germinal cells at all stages, from primary spermatocytes to mature spermatozoids	Rat
	TrkA	Spermatozoa and spermatids (Adults) Leydig cells (Newborn rats)	
	p75	Spermatogonia (Adults) Cellular layer that surrounds the seminiferous tubules (Newborn rats)	
[34]	NGF/TrkA/p75	Leydig cells, Sertoli cells, and spermatogonia at various stages Epithelial cells, stromal tissues of the caudal epididymis, and seminal vesicle	Adult Japanese monkey
[35]	NGF	Ejaculated sperm/sperm head and tail	Bovines
	TrkA	Ejaculated sperm/acrosomal cap, nucleus, and tail regions	
[36]	NGF	Leydig cells, pachytene and preleptotene spermatocytes, and elongated spermatids	Golden hamster
	TrkA	Sertoli cells	
	p75	Ubiquitous distribution in the testis	
[37]	NGF, TrkA, and p75	Testis, prostate gland, and seminal vesicle	Rabbits
[38]	NGF	Prostate/middle piece of ejaculated sperm	Llamas
	TrkA	Epithelial and muscular cells of testis, epididymis, bulbourethral glands, prostate/epididymal sperm, and middle piece of ejaculated sperm	
[50]	NGF, TrkA, and p75	Fetal testis between 14 and 19 wk of gestation	Human
	NGF	Sertoli and interstitial cells	
	p75	Peritubular cells	
[51]	NGF, TrkA, and p75	Adult human testis: prenatal testicular development Leydig cells (fetal and adult human testes)	Human
[52]	NGF and TrkA	Human sperm (proteins)	Human
	TrkA	Spermatozoa (mRNA)	

### 2.3. NGF in the Male Reproductive System of Humans

#### 2.3.1. Expression Sites

In humans, the expression of mRNA for NGF, the high-affinity receptor TrkA, and the low-affinity p75 receptor were confirmed in fetal testis between 14 and 19 wk of gestation. NGF was mainly expressed in Sertoli and interstitial cells, whereas the peritubular cells were the site of p75 expression, indicating a possible regulatory role for NGF in the proliferation and survival of germ and peritubular cells [50]. Müller et al., 2006, identified, through immunohistochemistry, NGF, p75, and TrkA in the prenatal and adult human testicles, where the Leydig cells were the predominant expression sites [51] (Table 1). The first evidence of NGF’s presence in ejaculated human semen came from Li et al., 2010 [52]. They explored the presence and expression levels of NGF and TrkA in oligoasthenozoospermic, asthenozoospermic, and fertile men. The presence of NGF and TrkA proteins as well as TrkA mRNA was confirmed, which, according to the authors, suggests that any effect of NGF on sperm activity can be performed via a paracrine pathway. Moreover, the NGF concentrations in seminal plasma from oligozoospermic men were lower, but not in a significant way, compared to the asthenozoospermic and fertile semen. Additionally, the TrkA mRNA levels seem to be significantly lower in spermatozoa from oligoasthenozoospermic men than in those from asthenozoospermic and fertile men.

Recently, the expression of NGF and its receptors were evaluated in semen and sperm from fertile men and patients with infertility. The results suggest that physiological NGF levels most likely bind primarily to TrkA and sustain sperm, while elevated NGF levels negatively impact sperm quality by activating the p75 receptor. As mentioned in animal models, the balance between p75NTR and TrkA may serve as a possible biomarker for sperm quality [53].

#### 2.3.2. Impact of NGF on Sperm Parameters

Abdulrahman et al., 2019, attempted to elucidate the relationship between NGF, sperm parameters, and seminal antioxidant capacity [54]. Hence, NGF levels were measured in the blood and seminal plasma of asthenozoospermic, oligoasthenozoospermic, and normozoospermic males. Although no significant correlations were detected between sperm parameters and serum levels of NGF, decreased NGF concentrations in serum and seminal samples of asthenozoospermic and oligoasthenozoospermic men were observed. It was further mentioned that the total antioxidant capacity (TAC) and the catalase (CAT) levels were significantly elevated in fertile compared to infertile groups. TAC activity showed a positive relationship with the total sperm motility in the seminal plasma of asthenozoospermic and oligoasthenozoospermic males, and the amounts of CAT in asthenozoospermic individuals showed a statistically significant correlation to the total sperm motility. The main limitation of the study is the low sample number which might be an issue affecting the results.

In 2012, Shi et al. investigated the role of NGF on human sperm motility parameters in vitro [55]. In the study, 20 male participants took part, and the spermatozoa used had more than 60% motility. The mean percentage of motile sperm was assessed in defined time intervals with NGF concentrations of 0.1, 1, and 10 μM. The results showed that NGF treatment at all concentrations, for 30 min incubation, significantly increased sperm motility parameters, in particular, average path velocity (VAP), average path velocity (VAP, mm/s), straight-line velocity (VSL, mm/s), curvilinear velocity (VCL, mm/s), beat-cross frequency (BCF, Hz), and linearity (LIN, %), compared to the control. The number of spermatozoa with grade A motility was increased and the number of spermatozoa with grade C and D motility was decreased, indicating that NGF promotes sperm motility in a time- and dose-dependent manner.

Similarly, in another study, Lin et al., 2015, explored the effects of NGF on sperm motility [56]. They incubated human spermatozoa with NGF (10 μM) for 30 min and they recorded motility parameters. They noticed a significant increase in the relative sperm movement distance. In addition, they investigated the optimal NGF concentration (0.1 μM, 1 μM, and 10 μM) in promoting sperm motility in vitro. The results showed that 1 μM and 10 μM NGF increased sperm motility to a great extent and the percentage of grade A spermatozoa. They concluded that NGF promotes the motility of human spermatozoa by increasing the movement distance and the percentage of grade A spermatozoa in a dose-dependent manner.

Asimakopoulos et al., 2021, investigated the effects of βNGF on the progressive motility and vitality of human spermatozoa, by adding the factor on the culture medium during sperm processing [57]. Two doses were examined, namely, 0.5 ng/mL and 5 ng/mL, with a 1 h incubation time. The results showed that βNGF significantly improves progressive motility and vitality of human spermatozoa compared to the control group, an effect that does not seem to be dose-dependent.

Another interesting topic of research was the use of NGF as a supplement in cryoprotection medium for human spermatozoa. Saeednia et al., 2015, examined whether the addition of different concentrations of NGF affects semen parameters, such as sperm vitality, motility, nitric oxide (NO) concentration, and DNA fragmentation, in spermatozoa from normozoospermic men after cryopreservation–thawing [58]. They found that NGF supplementation in semen cryopreservation medium at a concentration of 0,5 ng/mL significantly improved post-thaw vitality, motility, and NO amount and reduced DNA fragmentation. In asthenozoospermic samples, Saeednia et al., 2016, found similar results [59]. Treatment of frozen–thawed asthenozoospermic samples with 0.5 ng/mL exogenous NGF increased the percentage of total motility and decreased the percentage of immotile spermatozoa compared to untreated cryopreserved–thawed samples. NFG, at a concentration of 0.5 or 1 ng/mL, had also a positive effect on human sperm vitality.

#### 2.3.3. Therapeutic Implications of NGF in Male Infertility

The therapeutic significance of NGF on the male reproductive system looks promising, and although much research is still in the preclinical stage, there are several potential implications in spermatogenesis, testicular health, erectile function, sperm maturation, and motility. In particular, recent studies suggest NGF modulation could be beneficial for conditions such as Partial Androgen Deficiency of the Aging Male (PADAM), hypogonadism, and gonadal dysfunction induced by cancer treatments. NGF administration appears promising as an alternative treatment for hypogonadism and as a protective measure against gonadal damage from chemotherapy [45]. Moreover, supplementation with NGF in cryopreserved semen may enhance sperm viability and motility, while incubation with NGF in vitro may improve sperm vitality and progressive motility, potentially enhancing assisted reproduction outcomes [45,57].

However, more studies need to be carried out to understand the precise mechanisms of NGF actions in male infertility management and its use in assisted reproductive technologies. Future research should focus on exploring its clinical significance and practical application to fully establish the benefits and limitations with their use. Furthermore, it is crucial to expand our knowledge of NGF’s role in pathologic conditions of the male reproductive system and optimize its therapeutic use for male infertility.

### 2.4. Summary and Future Directions

In summary, the expression pattern of the NGF system in the male reproductive system varies according to the species and testicular development. In humans, NGF and its receptors are expressed in fetal and adult testis, principally in Leydig and Sertoli cells. NGF and its high-affinity receptor are present in human sperm, although in spermatozoa, only TrkA mRNA was detected. In the seminal plasma, the NGF protein concentration and TrKA mRNA expression appear to be different in fertile and infertile men.

**Figure 1:**
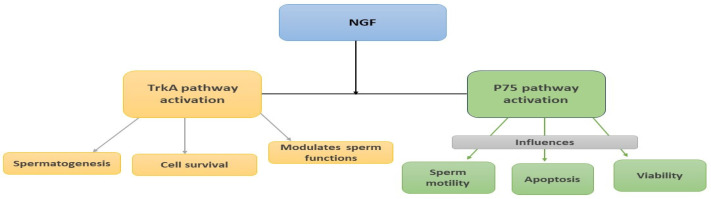
A schematic diagram showing the possible actions of NGF in the male reproductive system.

The NGF system seems to play pivotal roles, influencing processes such as spermatogenesis, morphogenesis, sperm function, and semen quality (Figure 1). TrkA activation by NGF promotes cell survival and modulates sperm functions, while p75 activation affects sperm motility, apoptosis, and viability. The balance between TrkA and p75 pathways determines sperm fate, with NGF levels influencing the interaction between these receptors. In addition, it seems that NGF increases in vitro motility possibly due to the activation of the MARK pathway while the supplementation of cryoprotection media with NGF can improve the post-thaw motility of frozen spermatozoa, offering potential benefits for assisted-reproduction techniques. Moreover, NGF has shown promise in therapeutic applications for male infertility and related disorders.

## 3. Erythropoietin (EPO)

### 3.1. Introduction

EPO belongs to the family of class I cytokines, and it is the key regulator of erythropoiesis. EPO is a glycoprotein with a molecular mass of 30.4 kDa composed of carbohydrate structures. The EPO gene encodes a protein precursor of 193 amino acids, and after cleavage of the amino acid leader sequence and posttranslational modifications, the final circulating peptide has 165 amino acids [60,61,62]. EPO folds into a compact globular structure consisting of 4-helical bundles [63] where the carbohydrate portion is required for the in vivo survival of the hormone [64,65]. Synthesis of EPO occurs mainly in the adult kidney and fetal liver. The peptide circulates in plasma with a half-life of 7–8 h and binds to the high-affinity receptor (EPOR) on the membrane of erythroid progenitor cells in the bone marrow [66].

The activation of the EPOR via ligand binding induces a dimerization and/or reorientation of EPOR monomers to a dimeric receptor structure [67,68,69,70,71,72]. The EPOR triggers various cellular processes essential for erythropoiesis. Upon interaction with EPO, the EPOR triggers the activation of multiple signaling pathways including Janus kinase/signal transducers and activators of transcription (Jak/STAT), which serves as the predominant pathway activated by EPOR [73,74,75], PI3K/Akt, and MAPK [76]. The biological consequences attributed to these pathways include viability/antiapoptosis, proliferation, differentiation, and maturation of erythroid progenitor cells. [76]. EPO may also act synergistically with other cytokines such as the stem cell factor (SCF), granulocyte-macrophage-colony stimulating factor (GM-CSF), interleukin 3 (IL-3), and IGF-1 to support the differentiation, proliferation, and survival of the erythroid progenitor cells [65,77,78].

The detection of EPOR in various nonerythroid cell types has led to a revision of the EPO biological function [79]. EPORs were shown to be present in the central nervous, cardiovascular, and reproductive systems [80,81,82,83]. Therefore, it has been proposed that the physiological role of the EPO/EPOR system may include angiogenic, vasculogenic, neurotrophic, and tissue protection functions [84,85,86,87,88]. However, it seems that the expression of the receptor differs in erythroid and nonerythroid cells. In erythroid cells, EPOR mRNA is expressed at sufficiently high levels for the protein dimerization that is needed for high-affinity EPO binding. On the contrary, mRNA expression in nonerythroid cells is low, and possibly these levels may not be sufficient to dimerize EPOR. Consequently, the low-affinity binding site for EPO may be a heterodimer composed of EPOR and another partner [89,90]. This may clarify the need for high levels of exogenous EPO for any effects in nonerythroid cells [66].

Regarding the expression and effects of EPO on the male reproductive tissues, several studies have been conducted and are summarized in Table 2.

### 3.2. Expression Patterns and Actions of EPO

The analysis in isolated Leydig cells of adult rats, conducted by Mioni et al., 1992, revealed the presence of specific binding sites for recombinant human erythropoietin (rHuEPO) and the existence of two distinct classes of receptors with different affinities for rHuEPO [91]. rHuEPO shares the same amino acid sequence as EPO, with a small difference in the carbohydrate moiety [92].

In contrast, in a subsequent study, Magnati et al., 2001, examined the expression of EPO mRNA in various testicular cell types, including Leydig, Sertoli, and peritubular myoid primary cell cultures of rats [93]. The findings indicated an exclusive presence of EPO transcripts in Sertoli and peritubular myoid cells, with no detectable indication in Leydig cells.

Moreover, Mioni et al., 1992, suggested that the addition of rHuEPO exerted a stimulatory effect on testosterone production proposing a direct influence of this protein on steroidogenesis in adult rat Leydig cells [91]. Foresta et al., 1995, proposed a mechanism by which rHuEPO exerts its stimulatory effect on Leydig cells; they assumed that its effect on steroidogenesis may involve protein kinase C activation and not the activation of adenylate or guanylate–cyclase systems or the Ca^2+^ flux [94]. Yamazaki et al., 2004, further suggested that EPO, by binding to a receptor on the surface of Leydig cells, can affect male reproductive function via a Jak-Stat pathway [95].

### 3.3. EPO in the Male Reproductive System of Animal Models

In mouse reproductive organs, a site of EPO production is the epididymis, where the site of the production is located in the interstitial space between the ductus epididymidis, suggesting that EPO supports the duct functions through paracrine mechanisms. In addition, a developmental and a transient, hypoxia-induced increase in epidermal EPO mRNA was recorded [96].

Further, in rats, Collares et al., 2012, constructed an EPO expression vector and transferred it to male rabbits via intramuscular injection; they examined the effects of nonviral EPO gene transfer and intravenous rHuEPO administration on sperm motility, viability, morphology, and concentration [97]. It appears that the EPO gene transfer and rHuEPO administration do not affect the sperm characteristics in the rat testis.

Akman et al., 2015, investigated the effects of Darbopoetin alpha (DP), a long-acting EPO analogue, on testicular and sperm parameters of adult rats who had received Doxorubicin (DXR) [98]. DXR is an anthracycline antibiotic that is used in cancer chemotherapy as it inhibits cancer cells [99,100]. Gonadotoxic effects of DXR disturb spermatogenesis in the testes and reduce both sperm concentration and motility [101,102]. As a result of the study, the administration of DXR in rats increased the levels of malondialdehyde (MDA), a widely used marker for oxidative lipid injury [103], in the testicular tissue. Further, a decrease in sperm vitality was observed. However, after DP administration, sperm mobility and viability were improved while sperm morphology was maintained, leading to the hypothesis that the protective effects of the EPO analogue are a consequence of its antioxidant properties.

### 3.4. EPO in the Male Reproductive System of Humans

The first indication of EPO expression in the human reproductive system came from Ascensao, 1983, who reported that a human testicular germ cell line produces significant amounts of EPO [104]. Later, it was reported that rHuEPO treatment improves sexual function in patients with end-stage renal disease [105]. In human Leydig cells, it was found that rHuEPO can directly influence the testicular steroidogenesis by stimulating testosterone production, an effect that seems to be independent of gonadotropin secretion [106].

The presence of EPO in human seminal plasma was examined by Temma et al., 2004 [107]. They found that EPO protein was constitutively present in the seminal plasma of fertile and infertile males in a range from 1.5 mIU/mL to 45.0 mIU/mL, suggesting that it probably origins from the prostate and the seminal vesicle. However, no association was observed between EPO concentrations and parameters such as sperm concentration, morphology, and concentration of leukocytes in semen. The presence of EPOR in human spermatozoa was evaluated in immunostained sperm samples, revealing over 90% positivity for the receptor [108].

**Table 2:** Expression of EPO/EPOR in the male reproductive system.

References	Expression of Protein/Receptors	Cell Type/Ort	Species
[91]	EPO (rHuEPO), EPOR	Leydig cells	Rat
[93]	EPO (mRNA)	Sertoli cells, Peritubular myoid cells	Rat
[95]	EPOR	Leydig cells	Rat
[96]	EPO	Epididymis	Mouse
[104]	EPO	Testicular germ cells	Human
[107]	EPO	Seminal plasma	Human
[108]	EPOR	Spermatozoa plasma membrane	Human

The in vitro effect of EPO on human sperm motility was further studied by Tug et al., 2010 [109]. In the experiments, washed spermatozoa from forty-two healthy volunteers were incubated with or without recombinant human erythropoietin beta (rEPO). Progressive and total motility showed a significant improvement with rEPO concentrations of 1, 10, and 100 mIU/mL compared to controls, setting the question whether this effect resulted from the antiapoptotic action of EPO or from improvemaent of motility per se.

Recently, Asimakopoulos et al., 2021, investigated the in vitro effects of an EPO analogue (epoetin alpha) on the motility and vitality of human spermatozoa selected after density gradient centrifugation and washing [110]. Forty-three volunteers were included in the research and two treatment groups were formed: one with low (10 mIU/μL) and one with high EPO concentration (100 mIU/μL). Each experimental group had its own paired control group. The incubation time was 1 h. The results of the study showed that treatment with EPO (low dose) significantly improved sperm’s progressive motility, nonprogressive motility, and vitality compared to controls. The high dose of EPO significantly improved progressive mobility and vitality but the nonprogressive mobility did not differ significantly from the controls. The results not only confirm the findings of Tug et al., 2010 [109], but in addition, Asimakopoulos and colleagues showed, for the first time, that treatment with EPO can significantly increase sperm vitality in vitro, in a not-dose-dependent way.

It is unclear exactly which pathways EPO uses to affect sperm motility and viability. It is hypothesized that EPO may bind to its receptor on the membranes of sperm, initiating processes that either strengthen mitochondrial activity or protect against oxidative stress and apoptosis, thus enhancing motility. Further research on the localization of EPO receptors on sperm and the pathways they trigger could shed light on how EPO enhances sperm function. Investigating these aspects could provide insights into both EPO treatment effects on sperm and sperm physiology overall [110].

### 3.5. Therapeutic Implications of EPO in Male Reproductive Disorders

The therapeutic significance of EPO on the male reproductive system lies in its potential to address certain reproductive disorders and improve fertility in men. Some therapeutic implications of EPO on the male reproductive system may include treatment of hypogonadism, its involvement in spermatogenesis, protection against hypoxia-induced damage in testicular tissue, enhancement of sperm cryopreservation, and its potential as a treatment for male infertility. While EPO shows promise as a therapeutic agent for various male reproductive disorders, further research is needed to clarify mechanisms and assess the safety and effectiveness of interventions, an understanding of these roles could result in novel diagnostic tools and treatments in the field of reproductive medicine.

### 3.6. Summary and Future Directions

**Figure 2:**
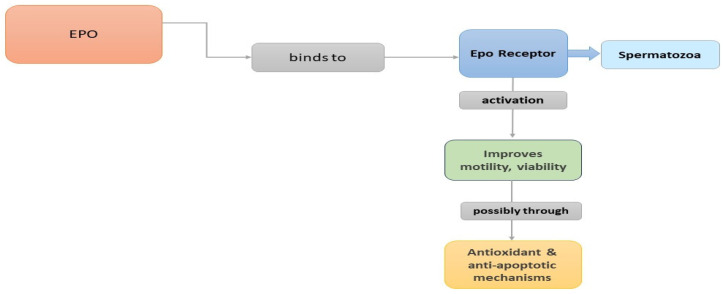
A schematic diagram showing the possible actions of EPO in humans as concerns motility and viability.

In summary, EPO is expressed in seminal plasma originated probably from the prostate and the seminal vesicles, while human testicular germ cells can also produce the peptide. In animal models, EPO receptors have been identified in Leydig cells, Sertoli cells, and the epididymis, while EPO receptors have been found in the plasma membrane of human spermatozoa. Many studies suggest potential roles of EPO in steroidogenesis, spermatogenesis, and sperm function. Moreover, it seems that incubation of spermatozoa with EPO increases motility. Probably, when EPO binds to the EPOR on the plasma membrane of spermatozoa, it induces a conformational change that activates signaling pathways improving sperm motility and viability, possibly through antioxidant and antiapoptotic mechanisms (Figure 2). Further investigation into the mechanisms underlying EPO’s effects on sperm function is needed to fully understand its therapeutic implications.

## 4. Insulin-like Growth Factor-1 (IGF-1)

### 4.1. Introduction

The insulin-like growth factors (IGFs) IGF-1 and IGF-2, also called somatomedins, are circulating proteins that share considerable structure homology and function with insulin [111,112,113]. They are members of the insulin-like growth factor (IGF) system which is the main regulator of cellular proliferation and somatic growth [114] and is composed, besides the two ligands (IGF-1 and IGF-2), of two types of receptors (IGF-1R and IGF-2R), six IGF-binding proteins (IGFBPs), and IGFBP proteases [115]. The IGFBPs are a family of multifunctional, high-affinity IGF-binding proteins. They play a central role in the bioavailability of IGF-1, which is a necessary factor for the IGF-1 actions. In humans, six IGFBPs (IGFBPs 1–6) are known; they share structural homology but differ in binding affinities for the IGFs, post-translational modifications, and susceptibility to proteolysis [116]. Several groups of IGFBP proteases, capable of cleaving specific IGFBPs and playing an essential role in their proteolysis, have been identified, including kallikreins, such as γNGF, prostate-specific antigen (PSA), cathepsins (such as cathepsin D), and matrix metalloproteinases [117].

Insulin-like growth factor-1 (IGF-1), also called somatomedin-C, is a low molecular weight peptide of 70 amino acids, synthesized mainly in the liver. IGF-1 acts as the main modulator of the growth hormone (GH) by regulating somatic growth but can also mediate anabolic actions independent of GH in several cells and tissues [118]. IGF-1 circulates as a complex bounded with specific IGFBPs. IGFBP-3 is the most abundant IGFBP and forms a binary complex with IGF-1 that is important for the movement of IGF in the extravascular space and its effects on peripheral tissues [114].

IGF-1 exerts its actions, such as growth, differentiation, proliferation, and mitogenic activities, mainly through the IGF-1 receptor (IGF-1R). IGF-1R is a glycosylated heterotetramer which belongs to the superfamily of the receptor tyrosine kinases. IGF–1R consists of two extracellular ligand-binding a-subunits and two transmembrane b-subunits linked by disulfide bonds. The b-subunit encodes an intracellular tyrosine kinase. Hormone binding activates the receptor kinase, leading to receptor autophosphorylation and tyrosine phosphorylation [119]. Some of the possible signaling pathways associated with receptor activation are PI3K/Akt/mTOR/GSK3β and Ras-MAPK [120].

Apart from other actions, IGF-1 is an important regulator of reproductive processes, influencing steroidogenesis, metabolism, cell proliferation, and differentiation [121,122]. In males, IGF-1 is necessary for the development of germ cells and normal morphology [121], while male mice with an IGF-1 null mutation are infertile [123].

In recent years, there has been growing interest in the investigation of IGF-1 actions in male reproductive physiology.

### 4.2. IGF-1 in the Male Reproductive System of Animal Models

#### Expression Sites and Actions

The IGF system has been well investigated in the male reproductive system of several animal species (Table 3). IGF-1 was found in bovine seminal plasma with similar concentrations to human seminal plasma, and the IGF-1R was present on ejaculated bovine spermatozoa, primarily localized to the acrosomal region [124]. In addition, the IGF-1R was found in mature sperm of rabbits, mainly located in the equatorial part, but its expression also takes place in the acrosomal region, midsection, and tail [125]. These results suggest that different species may have different subcellular IGF-1R localization in sperm cells. 

The presence of the IGF system in the seminal plasma and male reproductive tracts of mammals is thought to be linked to sperm motility and morphology, pregnancy rates, and fertility [126,127]. Ovensen et al., 1996, proposed that GH triggers the production of IGF-1 from Sertoli and/or Leydig cells, subsequently promoting the maturation of spermatozoa and leading to an increase in sperm motility [128]. In rats, Leydig cells have an IGF-1R, capable of modulating the synthesis of steroids [129].

Regarding the ability of IGF-1 to stimulate sperm motility, the study of Henricks et al., 1998 [124], in bovine species, showed that IGF-1 and IGF-2 were capable of maintaining sperm motility. In the study, IGF-1 and IGF-2 increased the velocity of sperm cells compared to the control. As mentioned by the authors, a potential mechanism that improves motility is through energy metabolism, as IGFs can increase glucose uptake, lactate production, pyruvate dehydrogenase activity, and glucose-6-phosphate conversion. Another possibility is that IGF-1 can act as an antioxidant. Lipid peroxidation and reactive oxygen species can damage the metabolism thus leading to a loss of sperm function and motility. So, if IGF-1 acts as an antioxidant, this would positively affect sperm viability [124].

Champion et al., 2002, studied the direct effect of GH and IGF-1 on the motility of equine spermatozoa in vitro [130]. They used recombinant bovine GH (100 ng/mL) or human recombinant IGF-1 (100 ng/mL) and compared their effects on motility and spermatozoa motion characteristics. They concluded that GH and IGF-1 significantly maintained spermatozoa motility compared to the control, whereas the spermatozoa motion characteristics were not significantly different from the controls.

The in vitro effects of IGF-1 on motility, membrane integrity, lipid peroxidation levels, and fructose uptake were also investigated by Selvaraju et al., 2009 and 2010, in frozen–thawed buffalo spermatozoa. IGF-1 was found to have a significant positive effect on sperm motility, and this improvement may be due to a reduction in lipid peroxidation [131,132]. Also, there was a significant increase in fructose uptake by the IGF-1 treated spermatozoa in a time-dependent manner, indicating that IGF-1 preserves motility by increasing fructose uptake. Additionally, a positive effect of IGF-1 on acrosin proteolytic activity was observed suggesting that IGF-1 maintains acrosomal membrane quality and a significantly decreased MDA concentration in spermatozoa [131]. Furthermore, the addition of IGF-1 prevented the deterioration of sperm mitochondrial membrane potential [132].

Zangeronimo et al., 2012, reported that the concentrations of IGF-1 in the seminal plasma of boars may increase the duration of sperm motility [133].

Mendez et al., 2013, attempted to determine the effects of IGF-1 in combination with vitamin E, as an antioxidant, on standard sperm parameters and antioxidant activity following the cryopreservation–thawing of boar semen [134]. The effects were evaluated after the addition of vitamin E (added before cryopreservation), with IGF-1 (added during thawing), on the quality of cooled boar semen. The results showed that the addition of IGF-1 in samples cryopreserved with vitamin E (300 μg/mL) led to higher sperm motility.

### 4.3. IGF-1 in the Human Male Reproductive System

#### 4.3.1. Expression of the IGF-1 System

In humans, IGF-1 expression was detected, via immunostaining, in Sertoli cells and primary spermatocytes, whereas, in the interstitium, some Leydig cells were also IGF-1 positive [135]. The presence of IGF-1 was also verified in human seminal plasma [121,122,136], probably of testicular [128] or epididymal [137] origin. In human ejaculates, the IGF-1 mean concentrations appear to vary from 2.7 nmol/L to 14.8 nmol/L, showing similarity with the mean values found in seminal plasma of bovine (18.8 nmol/L), porcine (2.3 nmol/L), and equine species (2.6 nmol/L) [138].

The IGF-1R was detected, via immunostaining, mainly in human secondary spermatocytes and in early spermatids. In addition, it was found, though with less positivity, in Sertoli cells, whereas a small number of Leydig cells showed an intense signal, suggesting that IFG-1 may act on the cell differentiation process through a paracrine mechanism [135]. IGF-1R mRNAs were found in the human germinal epithelium by Zhou and Bondy, 1993, whereas in the same study, no IGF-1 mRNA was detected in normal testicular tissue or the germinal epithelium [139]. In the human spermatozoon, the IGF-1R is located principally in the equatorial and, with lower density, acrosomal regions. This pattern seems to remain after the sperm capacitation process, indicating that the IGF-1R site does not follow a subcellular relocation [137]. Silvia Sánchez-Luengo et al. (2005) investigated IGF-1R expression in spermatozoa and seminal plasma from fertile and infertile men, where the presence of the IGF-1R was detected in seminal plasma from both fertile and infertile subjects but was not identified in the sperm of patients with a history of fertilization failure [136].

Regarding the IGFBPs expression in human testis, mRNA for IGFBP-2 was expressed in Leydig and Sertoli cells; IGFBP-3 mRNA was detected in the endothelium of testicular blood vessels; and mRNA for IGFBP-4 was present in Leydig cells, interstitial connective, tissue and endothelium. Moreover, IGFBP-5 mRNA was detected in connective tissue and in small amounts in Leydig cells, and IGFBP-6 mRNA was found in a number of peritubular cells and in the interstitial section [139]. Human seminal plasma contains IGFBP-1, IGFBP-2, IGFBP-3 fragments, IGFBR-4, IGFBP-5 protease, and PSA [128,138,140].

#### 4.3.2. Mechanisms of Action of IGF-1 in Sperm Physiology

IGF-1 signaling in the male reproductive system is essential for spermatogenesis, steroidogenesis, and overall reproductive function. Upon binding of IGF-1 to IGF-1R, downstream signaling cascades are initiated, leading to the activation of various intracellular pathways, including the PI3K/Akt and MAPK pathways [120], both associated with proliferation, differentiation, metabolism, and cell survival [141].

The PI3K/Akt pathway plays a crucial role in mediating the effects of IGF-1 on spermatogenesis. Activation of Akt promotes cell survival, proliferation, and metabolism, which are essential for germ cell development. Additionally, Akt regulates the expression of key transcription and growth factors involved in spermatogenesis. Moreover, the MAPK pathway, in response to IGF-1 stimulation, impacts germ cell proliferation and differentiation by activating ERK1/2, which exerts control over gene expression and cell cycle progression during spermatogenesis. [142]

However, it seems that IGF-1 also signals via the JAK-STAT pathway, which provides further intracellular crosstalk and cell-specific effects of IGF-1. Whether this signaling pathway is significant in testicular function and development remains uncertain, but the presence of STAT proteins in human sperm structures suggests potential unique functions [141].

The IGF-1R also plays a pivotal role in sperm capacitation and function. Notably, phosphoproteomics analysis identifies the IGF-1R as one of the most enriched tyrosine phosphorylation kinases associated with up-regulated phosphorylation substrate sites during sperm capacitation [143]. The increase in tyrosine phosphorylation serves as an indicator of sperm capacitation, acrosome exocytosis, and hyperactivated motility, suggesting the potential of targeting the IGF-1R-mediated tyrosine phosphorylation pathway for improving sperm functions in infertile men [143,144].

Understanding the role and regulation of IGF1R in sperm physiology is crucial for advancing research and developing therapeutic interventions aimed at improving male reproductive health.

#### 4.3.3. Impact of IGF-1 on Sperm Capacitation, Motility, and Vitality

The direct actions of IGF-1 and IGF-2, as well as those of IGFBP-2 and intact IGFBP-3, on human sperm motility parameters in vitro were first analyzed by Miao et al., 1998 [145]. Biological material was obtained from 15 healthy individuals. Motile spermatozoa were selected with the “swim-up” technique, and the samples were incubated for 10, 30, and 60 min with either IGF-1 (50 ng/mL), IGF-2 (50 ng/mL), or IGFBPs (125 ng/mL). The measured sperm parameters were curvilinear velocity (CV), progressive velocity (PV), linearity (Ln), straightness (St), amplitude of lateral head movement (ALH), and beat frequency (BF). The results showed that the addition of IGF-1 for 60 min reduced the mobility parameters while the addition of IGFBP-3 led to an increase in sperm motility. When IGF-1 was added together with IGFBP-3, these changes were no longer statistically significant. IGF-2 and IGFBP-2 showed no statistically significant changes for any of the incubation times.

Recently, the effects of IGF-1 on progressive motility and vitality were investigated by Asimakopoulos et al., 2021. Forty-three volunteers provided semen samples, and the spermatozoa were incubated for an hour (37 °C) in medium supplemented with IGF-1. Two concentrations of IGF-1 were tested: 100 ng/mL and 1000 ng/mL. IGF-1 in both concentrations significantly enhanced the progressive motility and vitality in comparison to the controls, whereas the effect of IGF-1 on vitality seems to be dose-dependent [57]. An additional interesting result of the research was that the factors IGF-1 and NGF were compared to each other in terms of their effect on progressive motility and vitality, and it appears that IGF-1 is more effective than NGF, in the concentrations used in the study.

As highlighted in the case of a patient with primary infertility and severe oligoasthenoteratozoospermia (OAT), the use of intradermal injections of IGF-1 led to a strong improvement in total sperm concentration and progressive motility [146].

Colombo and Naz 1999 detected IGF-1 protein in seminal plasma of both fertile and infertile subjects [122]. They found that IGF-1 levels were significantly correlated with the total sperm count whereas low IGF-1 levels were associated with oligozoospermia.

In more recent research, Lee et al., 2016, tried to elucidate the role of IGF-l in male fertility and to determine the relationship between IGF-1 concentrations in serum and seminal plasma [147]. They obtained data from 79 men separated in four groups based on their semen parameters: normozoospermia, asthenozoospermia, teratozoospermia, and a combination of two or more abnormal parameters. Seminal plasma and serum concentrations of IGF-1 were determined for each subject. The groups with abnormal sperm parameters exhibited significantly lower levels of serum IGF-1 compared with the normozoospermic group. Seminal plasma IGF-1 levels, however, did not differ significantly between the groups. A limitation of the study was that the participants in the normozoospermic group were also infertile.

The relation of seminal cotinine and IGF-1 levels in smokers with idiopathic OAT (iOAT) was studied by Hassan et al., 2008 [148]. In infertile smokers, significantly lower IGF-1 levels were observed, followed by infertile nonsmokers. The fertile smokers had the second-highest seminal IGF-1 levels compared to the controls. Seminal IGF-1 had a significant positive correlation with motile sperm, but seminal cotinine was significantly negatively correlated with both seminal IGF-1 and sperm motility. It is likely that smoking effects on sperm parameters could be mediated by a reduction in seminal IGF-1, and this reduction is correlated with infertility.

It is interesting to note that IGF-1 is one of the growth factors found in the follicular fluid (FF) that contribute to various aspects of follicular development and oocyte maturation. EPO and NGF, on the other hand, are not typically found in significant quantities in FF. FF is a complex mixture of substances surrounding the oocyte that appears at the time of fertilization indicating that there is some role of the fluid in the oocyte–sperm interaction [149]. Previous studies have revealed that FF affects various aspects of spermatozoa kinetics and properties, including motility stimulation, induction of the acrosome reaction, attracting sperm to the site of fertilization, and facilitating penetration of the oocyte [150,151]. IGF-1 has been associated with spermatogenesis and sperm function, suggesting that its presence in FF could influence sperm motility and fertilization processes. Further research is needed to fully understand the role of IGF-1 in FF and its impact on male fertility.

**Table 3:** Expression of the IGF-1 system in the male reproductive system.

References	Expression of Protein/Receptors/IGFBPs	Cell-Type/Ort	Species
[121,122]	IGF-1	Seminal plasma	Human
[124]	IFG-1/IGFR-1	Ejaculate sperm/Sperm cells— acrosomal region	Bovines
[125]	IGFR-1	Mature sperm—equatorial region, acrosomal region, midsection, and tail	Rabbits
[129]	IGFR-1	Leydig cells	Rat
[135]	IGF-1	Sertoli cells, primary spermatocytes, Leydig cells	Human
	IGFR-1	Secondary spematocytes, early spermatids, Sertoli cells	
[136]	IGF-1/IGFR-1	Ejaculates	Human
[137]	IGFR-1	Sperm cells—equatorial region, acrosomal region (lower density)	Human
[138]	IGF-1/IGFBP-2, IGFBP-4	Ejaculates	Human
[139]	IGFR-I	Germinal epithelium	Human
	IGFBP-2, IGFBP-3 IGFBP-4, IGFBP-5, IGFBP-6	Normal testicular tissue/germinal epithelium	
[140]	IGFBP-2, IGFBR-4, IGFBP-3, IGFBP-5, PSA	Seminal plasma	Human
[152]	IGFR-1	Preimplantation embryos	Human

#### 4.3.4. Therapeutic Potential of IGF-1

IGF-1 holds therapeutic promise for various aspects of male reproductive health, including spermatogenesis, testicular function, testosterone production, and sperm quality. Despite ongoing research into its clinical significance, initial findings suggest that IGF-1 supplementation may potentially improve sperm quality and fertility in men with reproductive issues. Understanding how IGF-1 affects sperm production and testicular function may lead to personalized medicine approaches and new therapeutics by focusing on the IGF-1 pathways. However, the therapeutic use of IGF-1 in the male reproductive system is still an active area of exploration, and further studies are needed to determine its efficacy and safety in clinical settings. Research on IGF-1 is clinically and translationally significant as it offers potential solutions to male infertility and other reproductive diseases.

### 4.4. Summary and Future Directions

**Figure 3:**
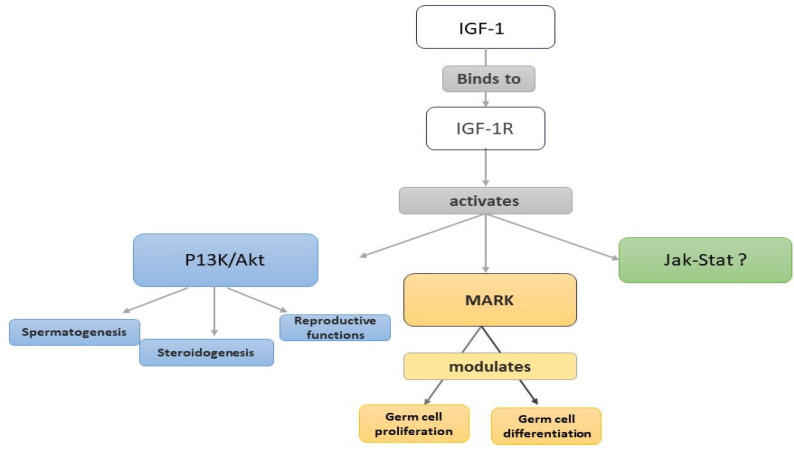
A schematic diagram of the possible mechanisms of actions of IGF-1 in the male reproductive system.

The available data lead to the notion that IGF-1 in humans is expressed mainly in Sertoli cells and is present in seminal plasma, while the IGF-1R is expressed in germinal epithelium and the equatorial region of spermatozoa. IGF-1 contributes to cell development and maturation of spermatozoa and supports testicular function, sperm production, and sperm quality (Figure 3). The levels of IGF-1 in the seminal plasma varies, being lower in infertile men or in smokers with OAT. Treatment with IGF-1 has a positive effect on sperm motility and vitality, and it seems that IGF-1 modulates sperm motility through mechanisms such as energy metabolism, antioxidant activity, and maintenance of mitochondrial membrane potential. Research on IGF-1 in the male reproductive system not only enhances our understanding of male fertility and reproductive health but also offers potential therapeutic avenues for addressing male infertility and reproductive disorders. Targeted treatments based on IGF-1 pathways hold promise for improving fertility outcomes and developing personalized interventions for men facing reproductive challenges.

## 5. Conclusions

This review highlights the significant roles of NGF, EPO, and IGF-1 in male reproductive physiology. These growth factors are expressed in the human reproductive system and are implicated in the maturation, differentiation, and survival of germinal and somatic cells. Additionally, they exhibit antioxidant and antiapoptotic actions. The available data indicate that NGF, EPO, and IGF-1 positively influence sperm motility, and lower levels of NGF or IGF-1 in seminal plasma are associated with infertility. Treatment of semen samples with these growth factors has shown promising results, particularly in terms of motility, suggesting their potential to improve the outcome of sperm processing techniques.

However, the mechanisms underlying their actions have not been fully clarified yet. While progress has been made in understanding their involvement, evidence gaps persist, necessitating further research to advance the field. For NGF, further studies are needed to elucidate the precise signaling pathways and molecular interactions through which it influences reproductive functions. Understanding species-specific variations in NGF expression is crucial for comprehensively addressing male reproductive health. Regarding EPO, further investigation into the mechanisms underlying its effects on sperm function is necessary to clarify its therapeutic implications. Additionally, understanding the physiological roles of the EPO/EPOR system in the male reproductive system requires further exploration. For IGF-1, comprehensive studies elucidating its mechanisms of action and expression patterns within male reproductive organs are needed. Addressing these gaps will enhance our understanding of NGF, EPO, and IGF-1’s roles in male reproductive health, potentially leading to improved diagnostic and therapeutic interventions.

## Data Availability

No new data were created or analyzed in this study. Data sharing is not applicable to this article.
